# Do Female Cancer Patients Display Better Survival Rates Compared with Males? Analysis of the Korean National Registry Data, 2005–2009

**DOI:** 10.1371/journal.pone.0052457

**Published:** 2012-12-26

**Authors:** Kyu-Won Jung, Sohee Park, Aesun Shin, Chang-Mo Oh, Hyun-Joo Kong, Jae Kwan Jun, Young-Joo Won

**Affiliations:** 1 Cancer Registration & Statistics Branch, National Cancer Control Institute, National Cancer Center, Goyang, Korea; 2 Department of Epidemiology and Health Promotion, Yonsei University Graduate School of Public Health, Seoul, Korea; 3 Molecular Epidemiology Branch, Research Institute, National Cancer Center, Goyang, Korea; 4 Cancer Early Detection Branch, National Cancer Control Institute, National Cancer Center, Goyang, Korea; The University of Texas M. D. Anderson Cancer Center, United States of America

## Abstract

**Background:**

Sex differences have been reported in the prognosis of certain cancers. In this study, we investigated whether Korean females display better survival rates compared with male patients for solid tumor sites.

**Methods:**

We analyzed data from the Korean National Cancer Incidence Database from 599,288 adult patients diagnosed with solid cancers between 2005 and 2009. Patients were followed until December 2010. We applied a relative excess risk (RER) model adjusting for year of follow-up, age at diagnosis, and stage at diagnosis.

**Results:**

For all solid cancer sites combined, women displayed an 11% lower risk of death compared to men (RER 0.89; 95% CI 0.88–0.90) after adjusting for year of follow-up, age, stage, and case mix. Women showed significantly lower RERs for the following sites: head/neck, esophagus, small intestine, liver, nasal cavities, lung, bone/cartilages, melanoma of skin, soft tissue, brain and CNS, and thyroid. In contrast, women displayed a poorer prognosis than did men for colorectal, laryngeal, kidney and bladder cancer. However, the survival gaps between men and women narrowed by increase in age; female patients over 75 years of age displayed a 3% higher RER of death compared with males in this age group.

**Conclusions:**

Female cancer patients display an improved survival for the majority of solid tumor sites, even after adjustment for age and stage. Age at diagnosis was the major contributor to the women’s survival advantage.

## Introduction

Sex is known to be an important factor in pathogenesis, diagnosis and treatment of cancers, and it has been an independent prognostic factor for several cancer sites.

There have been some studies that showed better cancer survival rates in women for lung [1], [2], [3], [4], CNS lymphoma [5], melanoma [6], [7], [8], and renal cell carcinoma [9]. Conversely, it has been reported that male patients with colorectal [4] and bladder cancer display a better prognosis over females [4], [10], [11]. When all cancer sites are combined, the survival rates for women are thought to be more favorable when compared with those of male patients [7], [8]. However, most studies have been conducted in western countries, and there were only few Asian data.

Women have a longer life expectancy in most countries [12], [13], and are more likely to be diagnosed with cancer at older age than men. If their asymptomatic cancers through cancer screening tended to be diagnosed more frequently in women, they could show better survival rates than men. Neither biological nor cultural factors clearly explain the survival advantage of women.

The purpose of this study was to investigate sex differences in survival among solid cancer patients after adjusting for age and stage of disease in a population-based setting using the Korean National Cancer Incidence Data for patients diagnosed from 2005 to 2009.

## Methods

### Study Population

Details of the history, objectives, and activity of the KCCR have been documented [14]. Briefly, the Korean Ministry of Health and Welfare initiated a nationwide, hospital-based cancer registry, the Korea Central Cancer Registry (KCCR), in 1980. The KCCR expanded cancer registration to cover the entire population under the population-based regional cancer registry program, and additional medical-record surveys have been conducted since 2003. The national cancer incidence reports for cancer patients diagnosed since 1999 have been published since 2005. The KCCR data from 1999 to 2002 have been published as Cancer Incidence in Five Continents [15], which reflects the completeness of the incidence data.

The current analysis included patients with solid cancers diagnosed between 2005 and 2009. We excluded non-melanoma skin cancer, and sex-specific sites, such as ovary, cervix, corpus, other gynecological sites, prostate, testis, and other male genital systems. Additional exclusion for breast cancer was done, as breast cancer is predominantly found in females and the sexes are known to differ with regard to biologically-based behaviors. The analysis was restricted to patients older than 20 years of age, since many features of childhood cancers differ from those of adult cancers. We included the first primary cancer only, and we excluded death certificate only cases.

Overall, a total of 599,288 adult patients with solid cancer were analyzed. Patients were followed until December 2010. The duration of survival for each case was calculated as the difference between date of initial diagnosis and date of death, loss to follow-up, or date of follow-up termination, whichever came first.

### Definitions and Statistical Analysis

The KCCR routinely collects data related to cancer, including information about demographic characteristics, location of the primary tumor, morphology, and stage at diagnosis.

The primary cancer was classified according to the International Classification of Diseases for Oncology, 3rd edition [16] and converted to the classification system used by the International Classification of Diseases, 10th edition [17]. Age at diagnosis was classified into four groups: 20–49, 50–64, 65–74, and 75 years or older. Stage at diagnosis was classified into four groups developed by Surveillance Epidemiology and End Results (SEER) [18]: localized, regional, distant, and unknown.

We applied a relative excess risk (RER) model to explain the sex difference between male and female [19], [20]. At first, we calculated relative survival rates, and these relative survival rates are then modeled using a generalized linear model with a Poisson error structure based on grouped data [19]. The hazard function, 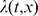 for a patients with characteristics *x* at time *t* is estimated as the sum of the known baseline hazard,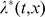, and the excess hazard due to a diagnosis of cancer,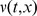. That is.
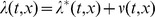



The model is written as
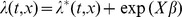



We included follow-up time in all models and restricted the analysis to the first 5 years of follow-up, as it is typically inappropriate to adopt proportional hazard assumptions for longer follow-up periods. To model the simultaneous effects of age and stage on patient survival, we applied a RER model adjusting for year of follow-up, age at diagnosis, and stage at diagnosis. All RERs given by the respective RER model were for women compared with men as the reference group. Models for each site were built separately.

To assess the effect of stage and age, we used a reduced model that excluded these covariates (Model 1). Model 2 estimated the RER adjusted for age, Model 3 estimated the RER adjusted for stage, and Model 4 considered both effects; 95% confidence intervals (95% CI) were also estimated. The regression analysis was performed on all ages combined and on four age groups. All analyses were conducted using SAS version 9.2.

## Results

Table 1 shows the characteristics of the study population and the stage distribution by cancer site. We analyzed a total of 599,288 solid cancer sites; 41.9% of these were in women. Most cancer types were more frequent in men, except for bladder and thyroid cancer. When we looked at 5-year relative survival, survival rates in women were higher than in men for 13 out of 20 cancer sites. For stomach, colorectal, liver, gallbladder, larynx, kidney, and bladder cancers, men’s survivals were better than women.

**Table 1:** Characteristics and stage distribution of the study population of Korean adults, 2005–2009.

ICD–10	Cancer site	Sex	N (%)	5 yr Relative survival	Age		Stage distribution (%)			
					Median	Mean	Localized	Regional	Distant	Unknown
C32	Larynx	Male	4,631 (93.5)	71.6	65	64.4	55.3	20.6	5.1	19.1
		Female	320 (6.5)	65.9	69.5	66.7	47.2	27.2	4.4	21.3
C15	Esophagus	Male	8,378 (92.0)	26.7	66	65.9	29.4	34.3	18.1	18.2
		Female	733 (8.1)	36.6	71	68.7	34.2	28.7	13.4	23.7
C67	Bladder	Male	11,527 (80.5)	77.6	67	65.9	65.2	12.4	3.4	19.1
		Female	2,798 (19.5)	69.2	71	69.1	60.6	12.8	4.8	21.8
C64–C66, C68	Kidney	Male	10,817 (80.5)	74.4	59	58.8	59.0	13.9	13.7	13.4
		Female	2,798 (19.5)	70.8	63	61.5	59.4	12.8	12.3	15.5
C22	Liver	Male	52,945 (75.4)	25.1	59	59.0	42.4	21.7	12.8	23.1
		Female	17,236 (24.6)	24.9	66	64.8	42.2	19.3	13.3	25.1
C00–C14	Head and Neck	Male	7,613 (73.6)	54.0	60	59.2	28.1	44.1	9.1	18.6
		Female	2,733 (26.4)	70.5	58	57.5	42.3	32.6	6.8	18.3
C33–C34	Lung, bronchus,and trachea	Male	57,031 (72.5)	17.0	68	67.2	17.6	26.6	37.1	18.7
		Female	21,658 (27.5)	23.8	69	67.4	19.3	20.4	41.1	19.2
C16	Stomach	Male	86,908 (67.0)	65.9	63	61.7	48.4	26.2	12.2	13.2
		Female	42,898 (33.1)	64.0	64	61.8	47.4	25.8	12.1	14.6
C30–C31	Nasal cavities	Male	739 (62.7)	50.6	60	59.2	32.9	37.2	7.3	22.6
		Female	439 (37.3)	55.5	66	63.4	38.5	26.4	11.4	23.7
C18–C21	Colon and rectum	Male	58,338 (59.1)	73.1	63	62.0	34.4	38.9	13.9	12.8
		Female	40,343 (40.9)	68.6	65	63.8	31.5	39.9	15.0	13.6
C17	Small intestine	Male	1,272 (57.6)	48.1	62	60.4	36.4	25.6	21.0	17.0
		Female	936 (42.4)	58.4	65	63.1	40.7	21.6	16.5	21.3
C47+C49	Soft tissue	Male	1,686 (56.6)	60.2	55	54.3	52.9	7.7	12.4	27.1
		Female	1,294 (43.4)	68.0	55	55.2	51.9	9.5	10.7	27.8
C25	Pancreas	Male	10,038 (55.4)	7.9	66	64.8	10.0	28.0	44.1	17.9
		Female	8,099 (44.7)	8.0	71	69.1	11.4	29.0	39.3	20.4
C40–C41	Bone and cartilages	Male	739 (53.8)	62.7	48	48.1	42.8	11.1	12.0	34.1
		Female	636 (46.3)	65.2	52	52.2	44.7	8.8	12.3	34.3
C70–C72	Brain and CNS	Male	3,270 (52.8)	37.4	52	52.5	62.0	3.6	2.3	32.1
		Female	2,923 (47.2)	41.6	56	55.4	60.1	2.9	2.6	34.4
C43	Melanoma of skin	Male	901 (50.0)	47.1	62	59.6	43.5	17.2	16.1	23.2
		Female	902 (50.0)	62.5	63	61.2	49.7	16.7	10.2	23.4
C23–C24	Gallbladder andbiliary tract	Male	9,295 (49.4)	27.0	67	66.6	23.7	37.1	19.7	19.5
		Female	9,541 (50.7)	25.2	71	69.6	23.0	33.0	23.2	20.8
C73	Thyroid	Male	15,786 (15.2)	99.3	46	47.2	36.6	48.9	1.9	12.7
		Female	87,933 (84.8)	99.8	47	47.3	43.9	42.7	0.9	12.6
	All other solid cancers	Male	6,110 (55.9)	31.2	65	62.5	14.8	9.2	18.3	57.8
		Female	4,816 (44.1)	31.5	68	64.8	12.4	7.3	20.9	59.4
C00–C80, except C44 andC50–C63	All solid cancers	Male	348,024 (58.1)	49.9	63	61.8	37.3	28.1	17.1	17.5
		Female	251,264 (41.9)	66.6	59	58.3	38.5	32.3	12.5	16.7

The median age of male cases was older than that of female cases for all solid cancer sites combined (63 vs. 59 years). However, female cases were older than males for the majority of cancer sites. This discrepancy is due to the case mix. The median age of those with thyroid cancer was the lowest among the 20 cancer sites and accounted for 35% of all solid cancers in females (87,933 cases among 251,264 female cancers). When thyroid cancer was excluded, male patients were, on average, 2 years younger than female patients (64 vs. 66 years, data not shown).

Distinct differences were observed in the stage distribution between men and women: those of regional origin accounted for 28.1% in men and 32.3% in women. For stomach, colorectal, liver, and kidney cancers, no differences in the stage distribution were observed between men and women. For head/neck, esophageal, small intestine, nasal cavity, bone/cartilage, melanoma of skin and thyroid cancers, women displayed a more favorable stage distribution than men.

Table 2 shows the RER analysis for 20 cancer sites. Four models are shown for the all-age analysis: Model 1 with follow-up years; Model 2 with follow-up year and stage; Model 3 with follow-up year and age; and Model 4 with follow-up year, age, and stage.

**Table 2:** Relative excess risk (RER) for solid cancer sites among Korean adults, 2005–2009.

ICD–10	Cancer site	RER for women			
		Model 1^a^ RER(95% CI)	Model 2^b^ RER(95% CI)	Model 3^c^ RER(95% CI)	Model 4^d^ RER(95% CI)
C32	Larynx	1.36 (1.08–1.71)	1.26 (0.99–4.59)	1.14 (0.90–1.45)	1.03 (1.01–1.92)
C15	Esophagus	0.87 (0.79–0.96)	0.90 (0.81–1.00)	0.80 (0.72–0.89)	0.83 (0.75–0.92)
C67	Bladder	1.57 (1.42–1.73)	1.47 (1.33–1.62)	1.34 (1.22–1.48)	1.24 (1.12–1.37)
C64–C66, C68	Kidney	1.15 (1.07–1.24)	1.21 (1.13–1.31)	1.00 (0.93–1.08)	1.10 (1.02–1.19)
C22	Liver	1.02 (1.00–1.05)	1.03 (1.01–1.05)	0.94 (0.92–0.96)	0.95 (0.93–0.97)
C00–C14	Head and neck	0.58 (0.53–0.63)	0.64 (0.58–0.70)	0.56 (0.51–0.62)	0.63 (0.57–0.68)
C33–C34	Lung, bronchus, and trachea	0.77 (0.76–0.79)	0.74 (0.73–0.76)	0.76 (0.75–0.78)	0.73 (0.72–0.75)
C16	Stomach	1.09 (1.07–1.12)	1.07 (1.05–1.09)	0.99 (0.97–1.01)	1.00 (0.98–1.03)
C30–C31	Nasal cavities	0.85 (0.70–1.04)	0.84 (0.69–1.03)	0.74 (0.60–0.91)	0.73 (0.59–0.90)
C18–C21	Colon and rectum	1.25 (1.21–1.28)	1.18 (1.15–1.22)	1.12 (1.08–1.15)	1.08 (1.04–1.11)
C17	Small intestine	0.76 (0.66–0.87)	0.80 (0.69–0.92)	0.65 (0.56–0.75)	0.70 (0.60–0.80)
C47+C49	Soft tissue	0.79 (0.69–0.92)	0.84 (0.72–0.97)	0.76 (0.66–0.88)	0.78 (0.68–0.91)
C25	Pancreas	1.01 (0.98–1.05)	1.03 (1.001–1.07)	0.95 (0.92–0.98)	0.97 (0.94–1.00)
C40–C41	Bone and cartilages	0.93 (0.76–1.14)	0.94 (0.77–1.15)	0.78 (0.63–0.95)	0.78 (0.64–0.95)
C70–C72	Brain and CNS	0.89 (0.83–0.95)	0.89 (0.83–0.95)	0.79 (0.77–0.85)	0.79 (0.74–0.85)
C43	Melanoma of skin	0.65 (0.55–0.78)	0.74 (0.62–0.88)	0.64 (0.54–0.76)	0.73 (0.61–0.87)
C23–C24	Gallbladder and biliary tract	1.11 (1.07–1.15)	1.07 (1.22–2.19)	1.03 (0.99–1.07)	1.00 (0.96–1.03)
C73	Thyroid	0.39 (0.30–0.51)	0.56 (0.45–0.69)	0.53 (0.41–0.70)	0.54 (0.44–0.67)
	All other solid cancers	0.94 (0.90–0.99)	0.88 (0.84–0.93)	0.88 (0.84–0.93)	0.83 (0.79–0.87)
C00–C80, except C44and C50–C63^e^	All solid cancers	0.92 (0.88–0.97)	0.96 (0.95–0.97)	0.91 (0.90–0.92)	0.89 (0.88–0.90)

a adjusted for year of follow up;

b adjusted for year of follow up and stage;

c adjusted for year of follow up and age;

d adjusted for year of follow up, age, and stage;

e adjusted for year of follow up, age, stage, and case mix.

According to analyses including participants of all ages, Model 1 showed that women displayed a significantly lower RER of death than did men for 10 out of 20 sites (head/neck, esophagus, small intestine, lung, melanoma of skin, soft tissue, brain/CNS, thyroid, all others, and all solid cancers), and men displayed a significantly lower RER than did women for 6 out of 20 sites (stomach, colon/rectum, gallbladder, larynx, kidney, and bladder). In Model 2, women displayed a significantly lower RER of death than did men for 9 out of 20 sites (head/neck, small intestine, lung, melanoma of skin, soft tissue, brain/CNS, thyroid, all others, and all solid cancers), and men displayed a significantly lower did than women for 7 out of 20 sites (stomach, colon/rectum, liver, gallbladder, pancreas, kidney, and bladder). In Model 3, women displayed a significantly lower RER of death than did men for 14 out of 20 sites (head/neck, esophagus, small intestine, liver, pancreas, lung, nasal cavities, bone/cartilage, melanoma of skin, soft tissue, brain/CNS, thyroid, all others, and all solid cancers), and men displayed a significantly lower RER than did women for only 2 out of 20 sites (colon/rectum and bladder). When we adjusted for follow-up years, age, and stage, the female advantage was almost identical to that in Model 3, with exception of larynx and kidney cancers, which favored male patients. All four models indicated an advantage for men in only two cancer sites (colon/rectum, and bladder).

When the analysis was performed by age group, women displayed a significantly lower RER than did men in most cancer sites, except for bladder (20–64 years) and colorectal (65–74 years) cancers, in the analysis for age groups younger than 75 years (Table 3). When the analysis was restricted to older patients (older 75 years), women displayed a lower RER than did men only for head/neck, nasal cavity, and lung cancers. Conversely, men displayed a survival advantage over women for 8 out (stomach, colon/rectum, liver, gallbladder, larynx, kidney, bladder, and all solid) of 20 cancer sites.

**Table 3:** Relative excess risk (RER) for solid cancer sites among Korean adults by age group, 2005–2009.

ICD–10	Cancer site	RER (95% CI) for women^a^			
		Age 20–49	Age 50–64	Age 65–74	Age 75+
C32	Larynx	0.93 (0.32–2.71)	0.67 (0.34–1.33)	0.69 (0.42–1.12)	1.53 (1.11–2.12)
C15	Esophagus	0.77 (0.53–1.12)	0.71 (0.57–0.89)	0.66 (0.54–0.81)	1.12 (0.96–1.31)
C67	Bladder	1.60 (1.01–2.54)	1.33 (1.04–1.70)	1.04 (0.86–1.24)	1.40 (1.21–1.61)
C64–C66, C68	Kidney	1.07 (0.86–1.33)	0.96 (0.83–1.11)	1.10 (0.97–1.26)	1.27 (1.09–1.47)
C22	Liver	0.81 (0.76–0.86)	0.87 (0.84–0.90)	1.03 (0.99–1.07)	1.11 (1.05–1.16)
C00–C14	Head and neck	0.63 (0.51–0.77)	0.57 (0.48–0.68)	0.58 (0.49–0.69)	0.75 (0.63–0.90)
C33–C34	Lung, bronchus, and trachea	0.70 (0.65–0.75)	0.60 (0.58–0.63)	0.73 (0.70–0.75)	0.87 (0.84–0.90)
C16	Stomach	1.01 (0.96–1.07)	0.92 (0.88–0.97)	0.94 (0.90–0.98)	1.12 (1.07–1.17)
C30–C31	Nasal cavities	0.70 (0.42–1.17)	0.84 (0.56–1.27)	0.79 (0.56–1.14)	0.59 (0.38–0.91)
C18–C21	Colon and rectum	0.90 (0.83–0.97)	0.99 (0.94–1.05)	1.07 (1.02–1.13)	1.27 (1.20–1.34)
C17	Small intestine	0.48 (0.30–0.78)	0.71 (0.53–0.95)	0.66 (0.52–0.84)	0.84 (0.64–1.08)
C47+C49	Soft tissue	0.95 (0.74–1.22)	0.52 (0.38–0.71)	0.76 (0.57–1.02)	1.01 (0.72–1.41)
C25	Pancreas	0.94 (0.83–1.05)	0.94 (0.88–0.997)	0.95 (0.90–1.00)	1.03 (0.97–1.10)
C40–C41	Bone and cartilages	0.82 (0.58–1.16)	0.81 (0.54–1.19)	0.84 (0.54–1.29)	0.62 (0.38–1.02)
C70–C72	Brain and CNS	0.78 (0.68–0.90)	0.73 (0.65–0.83)	0.85 (0.74–0.97)	0.85 (0.71–1.03)
C43	Melanoma of skin	0.88 (0.62–1.25)	0.58 (0.42–0.79)	0.75 (0.54–1.05)	0.77 (0.48–1.24)
C23–C24	Gallbladder and biliary tract	0.89 (0.76–1.04)	0.97 (0.90–1.04)	0.97 (0.91–1.03)	1.07 (1.01–1.15)
C73	Thyroid	NA	0.37 (0.22–0.61)	0.48 (0.34–0.68)	0.86 (0.61–1.21)
	All other solid cancers	0.80 (0.69–0.92)	0.71 (0.64–0.80)	0.77 (0.70–0.84)	1.01 (0.92–1.10)
C00–C80, except C44and C50–C63^b^	All solid cancers	0.88 (0.86–0.90)	0.80 (0.79–0.82)	0.88 (0.86–0.89)	1.03 (1.01–1.05)

a adjusted for year of follow up, age at diagnosis, and age;

b adjusted for year of follow up, age at diagnosis, stage, and case mix.

NA: Not applicable.

## Discussion

To our knowledge, this is the first study among an Asian population to use a population-based registry to investigate sex differences in cancer survival. We applied a RER model that adjusted for age and stage distribution using the KCCR database. We found that women displayed better survival rates for the majority of cancer sites; the exceptions were colorectal, laryngeal, kidney and bladder cancers. However, the female survival advantage diminished with age, with men displaying a survival advantage over women in patients aged 75 years or older.

For all the cancer sites combined, the excess mortality for females was 0.89 after adjusting for follow-up years, age, stage, and case mix. This difference was larger than those of a previous European study (4% lower RER) [8]. However, this big difference could be explained by case-mix with thyroid cancer which showed very high overall survival rate. Thyroid cancer accounted for 35% of all solid cancers in Korean females, female patients had a significant 46% lower RER than males. The observed sex differences in survival may be explained by tumor characteristics, such as distribution of morphologies [21], and difference in risk factors such as hormones [2], infections [22], [23], and chromosomal changes [24]. Differences in risk factor, particularly smoking, also constitute important factors. Smoking is known to be associated with a higher risk of death in cancer, and the smoking rate in Korean adults is 67.6% for males and 3.0% for females [25]. Previous studies [26], [27] have suggested that men are characterized by more co-morbid conditions at the point of cancer diagnosis than are women, and pre-existing chronic conditions may contribute to sex differences in survival rates.

We observed lower survival rates for women in cases of colorectal, laryngeal, bladder, and kidney cancer. For bladder cancer, we observed a RER of 1.24 for females following the adjustment for follow-up years, age, and stage. Recent analyses indicated poorer survival rates for women in the USA [28] and Europe [7], [8]. The disparity between sexes may represent differential levels of exposure to tobacco in addition to differences in the effects of genetic, anatomical, hormonal, and environmental factors [10]. For laryngeal cancer, our results are consistent with those of previous studies [4], [8]. We observed significant disadvantage for females (RER 1.03, 95% CI 1.01–1.19) of laryngeal cancer in Korea. For USA and Europe, females had a higher RER over males, but showed statistically non-significant results.

We observed a higher RER 1.08 (95% CI 1.04–1.11) for females of colorectal cancer after adjusting for follow-up years, age, and stage. Previous data on colorectal cancer are controversial and show both higher [7] and lower RERs of death in female patients [8], [29].

In contrast to previous reports, we observed a higher RER 1.10 (95% CI 1.02–1.19) for females of kidney cancer in Korea. In the majority of previous studies, women displayed better survival rates [4], [8], [9], [30], [31], [32]. In one study investigating sex differences in kidney cancer, Woldrich et al [32] displayed a male prevalence of 62% among kidney cancer patients. Women displayed a better stage distribution and a higher incidence of stage I tumors (54%) compared with men. In Korea, male patients were dominant (81% of kidney cancer patients), and women cancer patients were, on average, 4 years older than males (63 vs. 59 years). However, no sex differences in stage distribution were reported. The survival rates of kidney cancer patients were reported to be superior in females (64.4% in males and 69.7% in females diagnosed between 1996 and 2000), but these findings have been contradicted by those of more recent studies (77.2% in males, 76.8% in females diagnosed between 2005 and 2009) [33]. These discrepancies may be due to a differential increase in the incidental diagnosis of kidney cancer. Further analysis will be required to fully explain these discrepancies.

To assess the effect of stage and age on the RER separately, we used reduced models that excluded age (model 2) or stage (model 3) covariate. For most cancer sites, the RERs for females in model 2 were much higher than those of model 4. Therefore, the age covariate seemed to play a role in the lower RERs of women. For liver cancer, we saw the reverse results, *i.e.,* the male advantage in model 2 turned into a female advantage in model 4. When we assessed the effect of the stage covariate, some cancer sites had higher RERs in model 3 than model 4, including colorectal, gallbladder laryngeal, lung, and bladder cancers. These cancer sites showed that stage distribution in men was more favorable than in women.

We have found that age at diagnosis was the major contributor to the women’s survival advantage. The RERs for women increased with age; female patients displayed a 3% higher RER of death compared with men older than 75 years for all solid cancer combined. Women’s advantage was most marked in 50–64 years, and reduced drastically in older than 75 years in most cancer sites. This female disadvantage with older age group was reported in recent European [7], [8] studies. Micheli at al [8] suggested that age at diagnosis might be a proxy for biological factors that changed more markedly in women than men as they got older.

We have some limitations in our study. First, we did not have information about histologic grade, co-morbidities, or risk factors including smoking. Thus, we adjusted only for age and stage, which may affect certain cancers. However, differences in the prevalence of smoking in women and men may have influenced the survival differences observed. Second, validation of the stage information is critical for a comparison of survival rates. Many registries routinely collect stage at-diagnosis data using the SEER summary due to the ease of data collection using medical records. Although the SEER stages are not as detailed as the stages defined by other systems, reports indicate that the SEER stages provide effective adjustments for stage at diagnosis [34]. However, the proportion of unknown staged cases reported in Korea was relatively high, rendering the data difficult to interpret and a direct comparison of RERs after adjusting for stage distribution difficult.

In conclusion, our analysis demonstrates that female cancer patients possess a lower mortality risk than do males after adjusting for follow up, age, stage, and case mix. Age at diagnosis was the main determinant of the female advantage with regard to cancer death. This was most evident in young and middle-aged patients, but it reversed in patients older than 75 years. Future studies should therefore focus on the etiological factors responsible for the systematically lower mortality risk among women.
